# Using a Proactive Telecare System to Support Independence, Health, and Well-Being in Older Adults: Feasibility Randomized Controlled Trial

**DOI:** 10.2196/82152

**Published:** 2025-12-31

**Authors:** Lauren Fothergill, Yvonne Latham, Niall Hayes, Carol Holland

**Affiliations:** 1 Division of Health Research, Faculty of Health and Medicine, Lancaster University, Health Innovation One, Lancaster, LA14AT, United Kingdom, 44 (0) 1524 65201; 2 Management School, Organisation Work and Technology, Lancaster University, Lancaster, United Kingdom; 3 Leeds University Business School, Faculty of Business, University of Leeds, Leeds, United Kingdom

**Keywords:** telecare, aging in place, older adults, feasibility study, acceptability

## Abstract

**Background:**

Proactive telecare offers services designed to reduce the occurrence of emergency situations by delivering proactive outbound calls and follow-ups and providing information and advice. By engaging regularly with users, proactive telecare may foster social connections with older adults and enable the detection of changes in needs. Telecare systems that promote active participation among older adults may also foster feelings of autonomy and self-management.

**Objective:**

This study aimed to (1) explore the acceptability and feasibility of delivering and evaluating a proactive telecare intervention to community-dwelling older adults prior to a potential effectiveness trial and (2) evaluate the proposed eligibility criteria and estimate the potential effect size of the impact of the intervention on health and well-being outcomes to inform sample size calculations for a future trial.

**Methods:**

An 8-week randomized pre-post feasibility study was conducted. Using a mixed methods approach, questionnaires and semistructured interviews were used to explore the feasibility and acceptability of the study. The proactive telecare system encouraged users to press an OK button once a day to confirm their well-being. If they did not respond, participants received a well-being check, and emergency contacts were notified if required. Outcomes associated with independence, health, and well-being were measured using standardized questionnaires, including health-related quality of life, mental health, and loneliness.

**Results:**

Thirty older adults were recruited, with 13 randomized into the intervention group and 17 into the control group. The mean (SD) age of the participants was 75.4 (5.2) years; 66.7% (20/30) of the participants recruited had more than one health condition. This study achieved high retention rates (30/33, 90.9%); however, the expression of interest rate was low (52/295, 17.6%), indicating that changes to recruitment strategies are required. Effect sizes for all quantitative outcomes were small (approximately 0.2). Participants demonstrated high acceptance of the intervention, with the primary benefit cited as providing reassurance and promoting autonomy. Proactive engagement encouraged self-regulation and allowed users to control the level of support received. Those who were socially isolated reported feeling less lonely because of having additional social contact. Most participants felt the intervention would be particularly beneficial if they were experiencing poor health that significantly affected their daily activities, suggesting it may be more suited to those with limited independence. Some participants expressed anxiety about using the technology, primarily due to a lack of understanding and uncertainty in their perceived need for the device.

**Conclusions:**

This proactive telecare system is feasible to deliver within a cohort of older adults living in the community. However, changes to recruitment approaches and implementation are needed to ensure acceptability and target numbers are achieved in a future effectiveness trial.

## Introduction

### Background

By 2030, the global population of older adults will reach 1.4 billion, which is set to increase to 2.1 billion by 2050 [1]. This demographic shift is likely to increase the number of people with health issues or disabilities, increasing the need for care of older people [2]. Promoting aging in place has become a key policy focus in the United Kingdom [3], as well as in other countries like Australia [4] and Canada [5]. This approach not only supports the health and well-being of older adults but also offers a cost-effective alternative to institutional care and helps address shortages in social care services [6]. Aging in place can be defined as “remaining living in the community, with some level of independence, rather than in residential care” [7, p. 133] or often referred to as independent living. However, aging presents physical, psychological, and social changes, such as functional decline, disability, widowhood, and increased risk of social isolation [8], which can reduce quality of life and independence [9]. Consequently, many older adults may need support to remain at home.

One approach to fostering independent living involves the utilization of telecare [10]. Telecare uses monitoring technologies, such as fall detectors and pendant alarms, to help older adults request assistance during emergencies [11]. Research has demonstrated that telecare may help promote well-being and health-related quality of life by providing a sense of security, reducing fear of falls, and increasing confidence [12,13]. However, previous research highlights concerns among older adults that telecare could be viewed as a cost-cutting strategy, potentially replacing in-person interactions [14]. This shift could contribute to greater social isolation and loneliness, factors that elevate the risk of all-cause morbidity and mortality [15]. Telecare is often implemented following incidents like falls, which can associate its use with aging and frailty [16,17]. Subsequently, telecare is frequently perceived by older adults as a final option [17], rather than a tool capable of actively promoting independence, health, and well-being.

Proactive telecare extends the support of fall detectors and pendant alarms by incorporating regular well-being monitoring [18]. This includes user-initiated check-ins via digital systems or outbound well-being calls [18]. In our previous research on proactive telecare, we found that regular engagement with older adults could facilitate early identification of emerging needs, acting as an early warning system [19]. We also found that allowing older adults to control the level of support provided through proactive telecare enhanced their perceived autonomy and improved access to social networks, particularly for those who were socially isolated [19]. Cund et al [20] similarly reported positive effects on the mental health of older adults using proactive telecare in Scotland. However, uncertainties remain about the full benefits of proactive telecare technologies to health and well-being [18].

This study focuses on a proactive telecare intervention, called *OKEachDay*, which allows users to check in daily by pressing an OK button or through outbound well-being calls. Evidence suggests that enhancing social support and increasing opportunities for social contact are effective strategies for promoting well-being and reducing loneliness [21]. This system tailors support based on the user’s level of independence [19], whereby if an older adult requires additional support, it is detected when they fail to press the OK button. Conversely, if the OK button is pressed, it indicates that no further support is needed. This approach offers users control over their care [19], potentially boosting autonomy and confidence.

Given the limited research on proactive telecare [18], there were several uncertainties about conducting an effectiveness study, including intervention acceptability, compliance with daily intervention use, willingness of participants to be randomized, outcome selection, and methods for outcome collection. As a result, a feasibility study was conducted to evaluate the study’s integrity for a future randomized controlled trial (RCT).

### Study Objectives

The objectives of this study are to (1) assess the acceptability and feasibility of delivering a proactive telecare system to community-dwelling older adults before a full effectiveness trial (eg, randomization, assessment measures, compliance with daily intervention use); and (2) evaluate the proposed eligibility criteria and estimate the potential effect size of the impact of the intervention on health and well-being outcomes to inform a sample size calculation for a future trial.

## Methods

### Ethical Considerations

Ethical approval for the study was granted by the Lancaster University Faculty of Health and Medicine Ethics Committee in September 2022 (ethics board approval number: FHM-2022‐1011-SA-1). Participants provided written consent before taking part, and data was deidentified. All participants were offered a £20 (US $22.14) shopping voucher as an appreciation for taking part in the study.

### Study Design and Procedure

A mixed methods approach, incorporating both quantitative and qualitative methods, was used to effectively address the research objectives. Participants were randomized in an equal 1:1 ratio into either the intervention group, which received the proactive telecare intervention immediately for a duration of 8 weeks, or a waitlist control group, which was offered the intervention after an 8-week delay. This design aimed to address ethical concerns related to withholding a potentially beneficial intervention. The 8-week trial duration aligns with prior studies examining the feasibility of technology-based interventions aimed at supporting independence in older adults [22,23].

Data for this study were collected between 2022 and 2023 in England. Individuals interested in participating were provided with an information sheet, outlining the study details and given the opportunity to ask questions before providing written informed consent. All participants completed a baseline survey with the lead researcher (LF) a week prior to commencing the trial (Multimedia Appendix 1). The survey assessed physical health, mental health, and other outcomes related to independence. Participants were randomly allocated into the two groups by the lead researcher using computer-generated random numbers. The same survey was administered again after participants had completed 8 weeks in the trial. Due to the nature of the intervention, neither participants nor the lead researcher could be blinded to group allocation.

### Participants and Recruitment

Older adults aged 65 years and above, who lived in their own home (not a care home) and who spoke English, were invited to take part. A sample of 30 participants was aimed for to adequately estimate the effect size (potential impact of the intervention) and test the feasibility of running a larger-scale trial [24].

Participants were recruited through various channels in the Northwest of England, including local councils, an older adult research volunteer group, and local community groups. Posters were distributed to community centers, and the lead researcher presented the research study at local older adult social groups. Staff at local councils aided in recruiting participants who had been identified as at risk of loneliness and social isolation. Potential participants contacted the lead researcher (LF) if they were interested in taking part in the study. The lead researcher then arranged a time to speak with each potential participant to explain what the research involved and to assess their eligibility and capacity to participate. Eligible individuals were provided with a participant information sheet and completed a consent form before commencing the study.

### Proactive Telecare Intervention

The system consisted of either a telephone or touchscreen device with an OK button for participants to press daily to confirm their well-being. Once consented to the study, participants receiving the intervention were contacted via telephone by proactive telecare staff. During this set-up call, each participant chose a preferred device (tablet or telephone), agreed on a time to press their OK button, and identified a nominated contact, often family or friends, who could be contacted if staff believed there were risks to the user. An automated reminder to press the OK button was played through the device 15 minutes before the participants’ agreed time. If the participant did not press their “OK” button by the agreed cutoff time, the call center team would attempt to contact the participant to confirm their well-being, which gave an opportunity for social interaction. If staff could not reach the participant via telephone, they contacted the participant’s nominated contact. In the event where nominated contacts could not be contacted, if staff believed there were critical risks to the user, emergency services were called.

Both devices have a button to press if the participant wishes to speak to the call center team, which could be used to call for help, to have a chat, or to raise other issues. Proactive telecare staff were available from 8 AM to 10 PM daily to support participants with general well-being and safety concerns. Proactive telecare staff are routinely trained in dementia awareness, suicide alertness, domestic abuse awareness, learning disability awareness, mental health awareness, and safeguarding. The service also offers additional courtesy calls to help people who may feel particularly isolated, which were offered to participants prior to taking part.

### Data Collection

#### Quantitative Data Collection

##### Participant Characteristics and Intervention Use

Demographic data (age, gender, education level, ethnicity, current or previous occupation, living arrangements, current levels of care, and health conditions) and participation rates were collected. The uptake of participants on initial approach and retention of participants recruited to the study were recorded. Participants’ engagement with proactive telecare was recorded to determine the feasibility of trial procedures and adherence with daily intervention use, which included the number of times a participant did not press their OK button, the number of calls between proactive telecare staff and participants, and the length of these calls.

##### Acceptability of Proactive Telecare

Participants in the intervention group were invited to complete an acceptability questionnaire to measure perceived usefulness, satisfaction, and ease of use of this proactive telecare using the senior technology acceptance model (STAM) 14-item scale (modified to fit the context of the intervention of interest) [25]. This measurement tool was used as it was designed to consider the needs of older adults, and it used the well-established technology acceptance model to underpin the questionnaire [26].

##### Health and Well-Being Outcomes

Standardized questionnaires were used to measure health-related quality of life, mental health, levels of loneliness, and perceived control and autonomy, reflecting key aspects of independence in older adults [27].

Health-related quality of life was measured using the short form-12 (SF-12) survey, due to its wide use and reliability [28]. The SF-12 measures 8 health domains, which are summarized into 2 scores, the Physical Component Summary and the Mental Component Summary. Mental well-being was measured using the Warwick-Edinburgh Mental Well-being Scale [29], which assesses hedonic well-being (happiness and life satisfaction) and eudaimonic well-being (positive psychological functioning and self-realization). It was chosen to capture positive well-being outcomes related to independence [27]. The Hospital Anxiety and Depression Scale was chosen to measure depression and anxiety [30], as it differentiates somatic symptoms that could be associated with aging as opposed to depression (eg, reduced appetite or poor sleep) [31]. The UCLA Loneliness Scale was used to measure loneliness [32], as the scale effectively measures participants’ *subjective* feelings of loneliness, rather than just social isolation. Quality of life was measured using the Quality of Life Scale (CASP-19 [Control, Autonomy, Self-Realization, and Pleasure, Quality of Life Scale in Older Adults]), which assesses control, autonomy, pleasure, and self-realization in older people [33]. This tool was chosen for its relevance to an older adult population and its focus on autonomy and control, which, our previous research suggests, improve with proactive telecare use [19].

### Qualitative Data Collection

Participants in the intervention group were asked to take part in a short semistructured interview upon completion of the trial. Participants from the control group who chose to use the intervention for 8 weeks after the initial waiting list period were also asked to take part in a semistructured interview. The semistructured interviews were used to explore the feasibility outcomes, including the acceptability of the proactive telecare intervention trial procedures (The interview guides can be seen in Multimedia Appendix 2). The interviews were conducted in person or over the phone if the participant preferred. All interviews were recorded with permission using an encrypted digital recorder and transcribed verbatim by the lead researcher and anonymized.

### Data Analysis

#### Quantitative Analysis

Baseline characteristics of the intervention and control participants were summarized using descriptive statistics. Effect sizes were calculated using Hedges *g* for future use in a sample size calculation. Hedges *g* was used as it is considered to be more accurate than Cohen *d* when analyzing small sample sizes [34]. Hedges *g* was interpreted using the recommended benchmarks of 0.2 for a small effect, 0.5 for a medium effect, and 0.8 for a large effect [34]. In keeping with the aims of a feasibility study, no inferential statistics were reported.

#### Qualitative Analysis

Interview data were analyzed using the framework analysis method [35] to facilitate comparisons between participants and align the data with our aims. The first author led the analysis, with another researcher coding 20% of the interviews and assisting in developing the framework matrix. The analysis began with both researchers reviewing and independently coding two initial transcripts. Codes were both deductive (using concepts from STAM and the research questions) and inductive (developed from the data). They discussed the codes for relevance and meaning, leading to the development of a preliminary analytical framework. A further two transcripts were coded by both researchers using the preliminary framework, taking care to note any new themes or codes that had not been previously included. Follow-up discussions resulted in revisions to the framework to incorporate new and refined codes. The lead researcher coded the remaining transcripts, refining the framework as new codes were developed. The themes were formed using existing concepts from STAM, for example, “perceived usefulness of the intervention” and “perceived ease of use.” The final analytical framework consisted of 13 concepts, organized into four categories, each defined by a brief description (Table 1).

**Table 1.** Framework analysis for feasibility and acceptability objectives.

Concept	Description
Acceptability and usability of proactive telecare	
Perceived usefulness of intervention	The extent to which the individual feels that the technology will support their independence, improve well-being or quality of life, make them feel safer, or promote a sense of control. Additional perceived benefits may include the ability to access assistance when needed (eg, contacting designated individuals), facilitating connections to social resources, and mitigating feelings of loneliness.
Perceived ease of use of intervention	The extent to which the individual believes that the technology is easy to operate, requires little mental effort, and is clear and understandable.
Technology anxiety	Refers to an individual’s hesitancy to engage with the technology, often stemming from unfamiliarity with its design, fear of making errors, or concerns about potential malfunctions.
Resistance to using technology	Refers to individuals who, despite the potential benefits of the technology, do not want to engage with it. This reluctance may be driven by financial constraints, a lack of perceived need, or reliance on alternative technologies or resources that already fulfill similar functions.
Improvements	Describes any improvements to the intervention that the participants suggest.
Appropriateness of eligibility criteria and study process	
Eligibility criteria	Describes identifying factors that highlight the appropriate people who may benefit from this technology.
Interest in taking part	Describes the participants’ reasons for wanting to take part.
Acceptability of trial procedure	
Study process	Describes participants’ views on the study procedures, including the randomization process, the clarity and adequacy of the information provided, and the study design.
Assessment measure	Describes participants’ views on completing the surveys, suggestions for additional outcome measures that could have been included, and any support required to complete the assessments.
Compliance	Describes the daily use of proactive telecare and any issues experienced.

## Results

### Participant Characteristics

The mean (SD) age of participants was 75.4 (5.2) years, and all participants were identified as White British (Table 2). The majority of participants were female (76.6%, 23/30), and more than half of the participants lived alone (63%, 19/30). A small proportion of the participants had informal carers (13.3%, 4/30), and just 10% (3/30) of participants currently used other telecare devices (in this case, pendant alarms). The majority of participants (93.3%, 28/30) had at least one chronic disease or health condition.

**Table 2.** Participant descriptive characteristics.

Characteristics	Intervention group (n=13)	Control group (n=17)	Total (n=30)
Age, mean (SD)	76.7 (5.9)	74.4 (5.1)	75.4 (5.2)
Gender, n (%)			
Female	10 (76.9)	13 (76.5)	23 (76.6)
Male	3 (23.1)	4 (23.5)	7 (23.4)
Lives alone, n (%)			
Yes	9 (69.2)	10 (58.8)	19 (63.3)
No	4 (30.8)	7 (41.2)	11 (36.7)
Living arrangement, n (%)			
Private accommodation	10 (76.9)	15 (88.2)	25 (83.3)
Housing association^a^	3 (23.1)	2 (11.8)	5 (16.7)
Education, n (%)			
No qualifications	4 (30.8)	3 (17.6)	7 (23.4)
Vocational qualification	4 (30.8)	4 (23.6)	8 (26.7)
GCSE^b^ or equivalent	0	2 (11.8)	2 (6.7)
A level or equivalent	1 (7.7)	3 (17.6)	4 (13.3)
Degree	3 (23)	2 (11.8)	5 (16.6)
Postgraduate	1 (7.7)	3 (17.6)	4 (13.3)
Has an informal carer, n (%)			
Yes	3 (23.1)	1 (5.9)	4 (13.3)
No	10 (76.9)	16 (94.1)	26 (86.7)
Diagnosed health condition, n (%)			
None	1 (7.7)	0	1 (3.3)
One	2 (15.4)	6 (35.3)	8 (26.7)
More than one	10 (76.9)	10 (58.8)	20 (66.7)
Prefer not to say	0	1 (5.9)	1 (3.3)
Current or previous occupation, n (%)			
Professional	7 (53.8)	5 (29.2)	12 (40)
Managerial	0	2 (11.8)	2 (6.6)
Clerical	1 (7.7)	4 (23.6)	5 (16.7)
Service and sales	4 (30.8)	1 (5.9)	5 (16.7)
Skilled agricultural	0	1 (5.9)	1 (3.3)
Trade work	1 (7.7)	4 (23.6)	5 (16.7)
Other telecare use (pendant alarm), n (%)			
Yes	2 (15.4)	1 (5.9)	3 (10)
No	11 (84.6)	16 (94.1)	27 (90)

a Housing associations provide affordable housing options, primarily for low- and moderate-income households.

b GCSE: General Certificate of Secondary Education, completed at age 16 years old in the United Kingdom.

### Recruitment and Retention Feasibility

**Figure 1.**
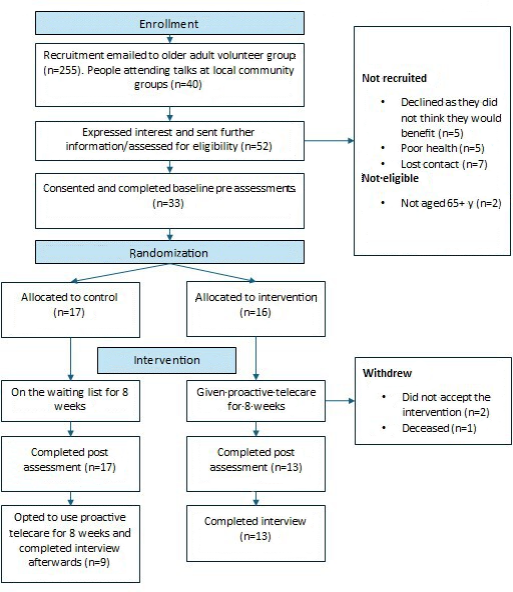
Consolidated Standards of Reporting Trials (CONSORT) flowchart of study recruitment, retention, and data collection.

Of the 295 individuals who received recruitment emails and attended an information session, 52 (17.6%) expressed initial interest. Of these, 50 (96% eligibility rate) were eligible, and 33 (66% recruitment rate) consented to participate. Reasons for nonparticipation included poor health (n=5), lack of perceived benefit from proactive telecare (n=5), and no response to follow-up emails (n=7). Among the 33 participants, 17 were randomized to the control group and 16 to the intervention group. A total of 30 participants completed the trial, yielding a 90.9% retention rate. Two intervention group participants withdrew, and one passed away during the study. Participant flow is summarized in Figure 1.

### Compliance With Daily Intervention Use

Participant engagement with the device was high, as most participants pressed their button daily or engaged with staff via phone. While almost all missed pressing their “OK” button at least once during the 8-week trial (with an average of 7 [range 1‐49] missed presses), the level of interaction remained consistent. The average number of calls between participants and proactive telecare staff was 10 (range 3‐57), with an average call length of 2 minutes and 27 seconds. Calls included participant-initiated contact or staff-initiated follow-ups after missed button presses. One participant did not press their OK button purposefully every day to receive a call from proactive telecare staff, as they felt socially isolated and wanted daily contact. For those requesting extra courtesy calls, the average call length was 4 minutes and 38 seconds.

### Health and Well-Being Outcomes

The health and well-being outcomes are presented in Table 3. In the intervention group, self-reported physical health from the SF-12 (Physical Component Summary) improved slightly compared to the control group (unadjusted between-group difference=4.92). Both groups showed a slight reduction in self-reported mental health (Mental Component Summary). Anxiety and depression levels remained stable in both groups, and quality of life decreased slightly in both. Mental well-being improved in the intervention group, while it decreased in the control group (unadjusted between-group difference=2.54). Loneliness increased in both groups. Effect sizes for all outcomes were small (approximately 0.2). With a small effect size of 0.2 and 80% power, a sample size for an RCT would be 150 participants, based on suggestions by Faul et al [36]. There were no missing data at the two time points, as the lead researcher either read the survey to participants or checked for missing responses during data collection. Two control group participants accidentally received the intervention due to human error by the telecare company. They were kept in the control group for outcome analysis to adhere to intention-to-treat principles [37].

**Table 3.** Health and well-being outcomes at the start (pre) and after 8 weeks of the intervention (post) and control trials

	Control group (n=17), mean (SD)			Intervention group (n=13), mean (SD)			
	Pre	Post	Within-group differences, mean (SD)	Pre	Post	Within-group differences, mean (SD)	Intervention group effect size (Hedges *g*)^a^
Health-related quality of life (SF-12^b^)							
PCS^c^	45.16 (10.38)	43.24 (10.69)	−1.87 (9.28)	39.15 (9.01)	42.08 (10.19)	3.05 (5.58)	0.305
MCS^d^	50.10 (11.35)	46.95 (12.25)	−3.15 (9.32)	46.92 (10.49)	43.62 (9.42)	−3.34 (6.08)	0.331
Mental well-being (WEMWBS^e^)	52.76 (11.26)	51.76 (11.68)	−1 (6.72)	46.38 (11.42)	47.92 (8.87)	1.54 (7.38)	0.151
Anxiety and depression (HADS^f^)	9.18 (7.34)	9.76 (7.28)	0.58 (3.89)	13.16 (7.03)	13.23 (6.76)	0.07 (3.82)	0.010
Loneliness (UCLA^g^)	29.47 (11.76)	31.47 (13.34)	2.00 (9.40)	38.69 (17.51)	40.69 (16.17)	2.00 (10.90)	0.119
Quality of life (CASP-19^h^)	42.71 [8.53)	41.12 [10.65)	−1.59 [7.87)	39.15 [8.41)	37.77 [7.59)	−1.38 [7.433)	0.172

a Hedges *g* presents the effect size for the intervention group.

b SF-12: short form-12.

c PCS: Physical Component Summary.

d MCS: Mental Component Summary.

e WEMWBS: Warwick-Edinburgh Mental Well-Being Scale.

f HADS: Hospital Anxiety and Depression Scale.

g UCLA: University of California, Los Angeles.

h CASP-19: Control, Autonomy, Self-Realization, and Pleasure, Quality of Life Scale in Older Adults

### Acceptability of Proactive Telecare

Both the quantitative technology acceptance survey and qualitative interviews indicated a generally positive perception of proactive telecare. Participants responded to 14 items assessing acceptability, using a Likert scale (1=strongly disagree and 10=strongly agree; see Table 4). On average, participants rated proactive telecare as useful (mean 7.3, SD 3.0) and agreed that it supported their ability to live independently (mean 7.7, SD 3.0). The system was also rated as easy to use (mean 9.5, SD 1.3). Participants generally disagreed with the statement indicating apprehension about using the technology (mean 3.1, SD 3.2). However, participants on average agreed that the cost of the intervention was a concern (mean 6.7, SD 3.7).

**Table 4.** Technology acceptance survey responses. Participants were asked to indicate their agreement with the technology acceptance statements below, using a Likert scale (1=strongly disagree, 10=strongly agree).

Technology acceptance statements	Scores, mean (SD)
Attitudinal beliefs	
Using proactive telecare enhanced your ability to live independently	7.7 (3.0)
You found proactive telecare useful in your daily activities	7.3 (3.0)
You like the idea of using proactive telecare	8.0 (2.4)
Control beliefs	
Proactive telecare was easy to use	9.5 (1.3)
You could complete a task using proactive telecare if there was someone to demonstrate how	9.1 (2.2)
Your financial status does not limit your activities in using proactive telecare	6.7 (3.7)
When you want or need to use proactive telecare, it is accessible to you	9.4 (1.5)
Technology anxiety	
You feel apprehensive about using proactive telecare	3.1 (3.2)
You hesitate to use proactive telecare for fear of making mistakes you cannot correct	2.7 (2.8)
Health conditions	
How are your general health conditions? (with 1 being very poor and 10 being very good)	6.7 (2.4)
How well are you able to concentrate? (with 1 being very uneasy and 10 being very easy)	7.8 (1.5)
How satisfied are you with your personal relationships? (with 1 being very unsatisfied and 10 being very satisfied)	8.3 (1.9)
How satisfied are you with the support received from friends and family? (with 1 being very unsatisfied and 10 being very satisfied)	8.4 (2.2)
How satisfied are you with your quality of life? (with 1 being very unsatisfied and 10 being very satisfied)	8.1 (1.5)

The findings from the quantitative acceptance survey were corroborated with the qualitative findings. Three themes were interpreted from the data: (1) perceived usefulness of proactive telecare, (2) perceived ease of use, and (3) technological anxiety and resistance.

### Perceived Usefulness of Proactive Telecare

Participants indicated that the most valuable aspect of the proactive telecare system was the reassurance provided by having a remote support network monitoring their physical and mental well-being, along with the ability to request help if needed.

*I would describe it really as a comfort blanket, you just know that it’s as though somebody’s looking out for you and I think that’s a nice feeling when you're getting older, just that you don’t want to be alone*.

[Participant 18]

Participants echoed the importance of proactive engagement in providing reassurance of safety. Participants also emphasized the benefit of being proactive in promoting self-initiation and self-regulation.

*It’s reassurance, isn’t it, I think it’s a psychological trigger. I think it’s a good thing, I really do*.

[Participant 12]

One participant highlighted the value of the flexibility of the intervention because users had choice and control over the level of support provided if they missed their OK button, in comparison to a pendant alarm where activating it indicates an emergency in an “all or nothing” approach to support.

*you press that [pendant alarm] for help, that’s like saying it’s an emergency, do I really need it? Just to say like in the morning yeah, I'm OK today, that’s better I think*.

[Participant 30]

While some participants felt they were slightly too young to require a proactive telecare system, several expressed surprise at the perceived benefits of having a remote monitoring team overseeing their well-being. They noted that, in the event of an incident such as a fall where the “OK” button was not pressed, the system would initiate a response to check on their well-being, which many participants found reassuring. However, a few were skeptical about the system’s effectiveness in promoting in-home safety and preferred alternatives like pendant alarms or mobile phones for requesting help. Approximately half of the participants suggested using a pendant alarm in conjunction with the proactive telecare system to enhance the ability to request help when needed.

*I do think that people who are in danger of falling need a falls alarm as well*.

[Participant 3]

Participants characterized the proactive telecare staff as friendly, empathetic, and supportive, contributing to a sense of being cared for and emotionally reassured.

*It was nice. It felt to me as if they really cared about me, it felt personal, I could feel as if that lady or that young man was ringing me because they were concerned about me*.

[Participant 4]

Two participants reported experiencing feelings of loneliness prior to their involvement in the study and subsequently chose to receive additional courtesy calls from the proactive telecare staff. These participants described forming positive interpersonal relationships with the staff and emphasized that the human contact provided through these conversations was more meaningful to them than the reassurance of safety alone.

*The people at [proactive telecare service] are beautiful people who are lovely, I think it has helped and, like I say, them ringing me twice a week, it’s really been nice. I’ll miss it really; you don’t feel as lonely*.

[Participant 10]

Although most participants declined the additional courtesy calls, as they believed their existing levels of social interaction were sufficient, participants recognized the potential value of such calls in offering social support to older adults experiencing isolation.

### Perceived Ease of Use

All participants reported that the proactive telecare intervention was easy to set up and use. Most opted to use the touchscreen device, while two participants preferred the telephone-based version. The telephone devices were installed in person by the proactive telecare staff, whereas the touchscreen devices were delivered by post, with setup instructions provided remotely via telephone.

*I'm a technophobe, I'm useless with things like that, but no it didn’t bother me at all. It was simple to use. I plugged it in the dining room and just did it every morning in the allotted time and it was just very, very, very simple to* use. (User talking about using the tablet)

[Participant 9]

Most participants reported that the device was not intrusive and was not burdensome, which was viewed as positive and facilitated the development of a routine for pressing the OK button. However, some participants stated that the requirement to engage with proactive telecare daily was cumbersome and became tedious, particularly when they forgot to press the button and subsequently received follow-up calls.

*I actually feel quite relieved, I haven't got to do it anymore [after the trial]. So perhaps I felt, it did tie me down - that I've got to remember to do it*.

[Participant 5]

Most participants reported occasionally forgetting to press their button but found the automated reminders helpful as a gentle prompt. They also appreciated the flexibility of being able to choose a time that suited their daily schedule, including the option to press the button up to 6 hours before the scheduled time.

*it didn’t matter if I did sleep a bit longer, if I didn’t wake up till nine o'clock I could still press it and it was alright*. (User’s cut off time was 10 AM).

[Participant 21]

This flexibility supported participants in remembering to press the OK button and enhanced the system’s accessibility by accommodating diverse daily routines.

### Technological Anxiety and Resistance

Although most participants found the technology easy to use, some reported initial apprehension when first engaging with proactive telecare, expressing fear of pressing incorrect buttons and making mistakes. One participant chose the telephone version of the device because they found it more familiar.

*I'm not good with a tablet, I thought at least with the telephone I know there were them three things and that’s all I needed to press*.

[Participant 11]

Some participants stated that their lack of understanding of how the technology worked enhanced their anxiety about making a mistake and that more comprehensive explanations would have been beneficial. For some, the unfamiliar design of the tablet contributed to feelings of confusion and apprehension.

*In the early days, I touched it in the wrong place to try and bring the screen back up again. And because I wasn't familiar with the screen, I touched the alert call. And then I couldn't see in my panic, how to cancel it. And, you know, felt really quite stupid*.

[Participant 5]

Some participants stated that they would have preferred a face-to-face explanation of the technology, noting that they learned more effectively through visual demonstrations. However, most were satisfied with the telephone-based introduction to proactive telecare.

Some participants reported that they would only consider adopting proactive telecare after experiencing functional decline, a decision influenced both by the cost of the device and by associations between the technology and aging or declining health.

*I'm only eighty and I can still get about, but somebody who couldn’t get out of the house or needed help, it would be ideal for them*.

[Participant 18]

Some participants felt that a perceived need for technological support was essential for engaging with the intervention. The presence of chronic conditions or disabilities was also seen as a key factor that could make individuals more likely to benefit from proactive telecare.

*I think it would be very handy for those who aren’t quite, very well. There is knowing that there’s a backup there if anything starts to go wrong*.

[Participant 17]

In contrast, some participants viewed the proactive telecare system as a potential precursor to using a pendant alarm, appreciating that it did not require constant bodily wear and that they did not yet feel “ready” to adopt a pendant.

One participant noted a desire to continue using the intervention but indicated that financial constraints, due to the cost of other telecare services, prevented them from affording both devices.

*I’d have liked to have kept it, you know, but now I've got this to pay for this pendant, it’s too expensive to have both*.

[Participant 13]

### Appropriateness and Acceptability of Trial Procedures

#### Eligibility Criteria

The inclusion criteria for this study were intentionally broad, based on the assumption that older adults aged over 65 years have diverse physical and emotional needs. When asked about their motivations for participating, most individuals expressed a general interest in contributing to research and a desire to give back to their local community. Additionally, some participants were motivated by curiosity about telecare technologies and a wish to explore potential personal benefits.

*I thought I'd like to test a system where I could make contact if I did inadvertently fall or, in any way become unsafe at home, and it came up*.

[Participant 2]

Participants supported the use of broad eligibility criteria, viewing the decision to adopt the technology as dependent on individual perceived need. They identified several circumstances in which proactive telecare could be beneficial, including for individuals who are housebound, living with chronic conditions or disabilities, have a history of falls, or experience limited social support. While some participants emphasized the benefits of the system for those living alone, others highlighted its value for individuals cohabiting with others, particularly if both people had chronic conditions.

*I think it fits with us quite well because we've both got problems and you don’t know if we’re going to finish up in hospital and then the other’s on their own all of a sudden, it’s an insurance*.

[Participant 13]

### Randomization and Assessment Measures

Most participants expressed satisfaction with being randomized into either the intervention or control group. However, a small number of participants preferred to be in the intervention group, so they could use the technology straight away. One participant, who reported feeling extremely isolated, expressed a strong desire to begin the intervention immediately.

Many participants reported that the trial questionnaires were acceptable and easy to complete. Nevertheless, a few participants described difficulty in answering some of the questions due to the subjectivity of some questions. One participant, for example, described challenges in answering questions that required recalling emotions or feelings experienced over the past few weeks.

*they asked you to remember the last week or the last four weeks. And at my age, you don't remember the last week or the last four weeks very clearly*.

[Participant 8]

Participants reported that the length of the survey was not burdensome, and the questions were deemed relevant to the study subject.

## Discussion

### Key Findings

This study assessed the acceptability and feasibility of evaluating a proactive telecare intervention in older adults living in the community. The trial sustained low dropout rates and successful collection of outcome variables. However, initial expression of interest in the study was low. Our mixed methods study suggested that proactive telecare was generally acceptable to participants; however, some participants indicated hesitancy as to whether this intervention was beneficial to them, so adaptations to the recruitment process should be explored. The trial procedures, including randomization and completing questionnaires, were feasible and acceptable to participants. Nevertheless, two instances of randomization contamination occurred, suggesting that revised procedures may be needed in a future full-scale trial.

Due to uncertainty about who would benefit from proactive telecare, the study adopted broad eligibility criteria: living in the community and being over 65 years old. This resulted in a high eligibility rate (96%), but only 17.6% of those contacted expressed interest. The low engagement may reflect the broad recruitment strategy, which targeted older adults generally who may not have perceived a need for telecare. Previous studies suggest that some older adults associate telecare with frailty, which may deter uptake [38,39]. Participants considered the criteria appropriate, emphasizing the importance of individual choice in assessing suitability. However, participants identified groups who may particularly benefit, including those who are housebound, have limited mobility, have chronic conditions, have disabilities, or lack social support. Future trials may be more effective if they target these specific populations.

Most participants found the study processes feasible and acceptable, with a high retention rate of 90.9%, consistent with findings from other studies involving older adults [40,41]. The high retention rate could be due to low participant burden imposed by the study. However, two participants withdrew due to issues with the technology. One participant was unable to establish a regular routine of use, and another was concerned about the risk of falling when standing to press the button, a concern also observed in other feasibility research involving frailer older adults at risk of falls trialing new technology [42]. Previous research suggests that telecare should integrate into individual contexts, routines, and abilities [43,44]. Providing additional support to assess users’ needs and preferences prior to implementation may help improve retention.

Many participants reported positive experiences of the study, including recruitment, randomization, and data collection methods. Many cited altruism as their reasons for taking part, which has been seen in other research involving older people [45]. While participants were generally comfortable with being randomized, a few expressed a preference for being placed in the intervention group rather than the control group. Two instances of contamination occurred, where participants allocated to the control group were provided with the intervention due to human error by the proactive telecare staff. Despite this, the participants remained in the control group to adhere to the intention-to-treat analysis [46]. Similar contamination issues have been noted in research evaluating interventions in primary care and community settings, where preventing contamination in RCT designs can be challenging [47]. In a full-scale trial, cluster randomization could be used, where groups of older adults are randomized instead of individual participants. This approach, within a defined setting such as in an assisted living environment where people live independently with additional support, could help overcome this issue.

Many participants found the outcome measures appropriate and the questionnaires easy to complete, though some noted the questions were subjective and at times difficult to answer. These findings indicate a need to optimize the measurement tools by simplifying and clarifying questionnaire items. Completing questionnaires with a researcher was viewed as helpful for clarifying meanings, suggesting researcher support may benefit future trials. Previous studies have also highlighted the importance of increased personal contact by researchers to support older adults taking part in research [45].

Only descriptive statistics were calculated to assess the feasibility of the RCT plan, as recommended for feasibility studies [48]. Standard deviations and effect sizes were calculated to be used in a future sample size estimation, as suggested by previous research [49]. Descriptive analyses found small improvements in mental well-being in the intervention group compared to the control group, which are similar to results that have been noted in other studies on telecare interventions [13]. These changes may reflect increased perceptions of safety and security, which in turn may improve perceived health and mental well-being [13]. In both groups, quality of life decreased, and loneliness scores increased; this may have been influenced by completing the surveys, which could increase participants’ awareness of loneliness or their quality of life. A larger-scale trial would be required to better understand the effects of proactive telecare on health and well-being.

This study found that proactive telecare was both acceptable and feasible for older adults. Participant engagement with the system was high, as all participants pressed their OK button daily or engaged with staff via phone. The most frequently reported benefit was a sense of reassurance, which has been noted in other studies on telecare [50]. While some participants did not feel they currently needed telecare due to their perceived independence, many still valued the reassurance that someone was checking in. Proactive engagement acted as a psychological prompt, encouraging self-regulation and allowing users to control the level of support received, as noted in our previous qualitative research on proactive telecare [19]. In contrast, existing literature on other telecare interventions, particularly monitoring technologies such as ambient sensors, emphasizes older adults’ concerns regarding privacy when using telecare and monitoring technologies [38,51]. The Farr Point report suggested that proactive well-being checks may be accepted by older people who are resistant to using other telecare devices due to associated stigma [18] or concerns about reduced perceptions of control and privacy [52]. This highlights the potential for proactive telecare interventions to promote autonomy and self-management, but further evaluation is required to fully understand this.

Most participants reported that the intervention was easy to set up and use and that it would be appropriate for older people who may not have experience in using similar technologies due to the simplicity of the system. There were a few participants who felt some apprehension toward the technology and would have benefited from having an in-person demonstration, which should be considered in a future trial. Wu et al [53] suggest that older adults often report a lack of knowledge of technology, which can result in apprehension. Product demonstrations are suggested to enable participants to trial and test out devices to gain further knowledge and confidence about the usability and usefulness of technologies.

For participants experiencing social isolation, the opportunity for social interaction was the most valued component of proactive telecare. Brief courtesy calls provided a sense of connection, with the supportive approach of staff helping to ease feelings of loneliness. However, the calls in this study lasted only around 5 minutes. In contrast, more intensive proactive telecare models, such as that examined by Cund et al [20], which involved longer, regular well-being calls, have been associated with improved mental well-being. A future RCT is warranted to evaluate the effectiveness of social support provided by proactive telecare on loneliness. Further research should also explore the optimal duration and nature of contact needed to reduce loneliness and promote well-being.

The findings from this study highlight key considerations for the future evaluation of proactive telecare. While participants valued the reassurance provided, those at higher risk may still require reactive devices for emergency support. This underscores the need for a flexible, person-centered approach, as a single technological solution is unlikely to meet the diverse needs of the older population. Future evaluations should consider how proactive telecare can be effectively integrated within local health and social care systems, including the use of other digital interventions like other telecare devices, telehealth, and telemedicine. This approach aligns with regional digital strategies in the United Kingdom, which aim to provide more preventive, proactive, and fully integrated telecare services [54]. Future evaluations should also consider how to offer proactive telecare beyond the research period, as some participants may have benefitted from continued use.

### Strengths and Limitations

A key strength of this study was its mixed methods design, which provided both quantitative outcomes and qualitative insights into participants’ experiences with proactive telecare. Standardized measurement tools were utilized to measure independent outcomes, which can be compared across the literature. However, several limitations should be noted. The sample lacked diversity, as all participants were White British, indicating a need for more inclusive recruitment in future trials. The 8-week duration may have been too short to observe full benefits, though longer follow-ups may affect retention. Additionally, the study focused on one type of proactive telecare, limiting generalizability.

### Conclusions

This study highlights key considerations for designing a future RCT of a proactive telecare system for older adults living in the community. The intervention was generally well-received, offering reassurance and, for socially isolated individuals, a sense of social connection. Study procedures were feasible and acceptable, though improvements in recruitment and implementation procedures are suggested to maximize uptake. The data from this study have provided valuable considerations for refining and justifying the design of a future effectiveness trial.

## Supplementary material

10.2196/82152 Multimedia Appendix 1 Quantitative survey questions

10.2196/82152 Multimedia Appendix 2 Qualitative interview guide

## Abbreviations

CASP-19: Control, Autonomy, Self-Realization, and Pleasure, Quality of Life Scale in Older Adults
RCT: randomized controlled trial
STAM: senior technology acceptance model
