# Supplementary material for: Using a Proactive Telecare System to Support Independence, Health, and Well-Being in Older Adults: Feasibility Randomized Controlled Trial
Source: JMIR Form Res. 2025 Dec 31;9:e82152. doi: 10.2196/82152 (PMC12755204; doi:10.2196/82152)
Supplement: Multimedia Appendix 1 [file formative-v9-e82152-s001.docx]

Supplementary File 1: Quantitative Survey Questions

| Demographic questions | | |
| --- | --- | --- |
| How old are you? |  | |
|  |  | Tick in this box |
| How would you describe your gender? | Male |  |
|  | Female |  |
|  | Other |  |
|  | Prefer not to say |  |
| How would you describe your ethnic background? | White |  |
|  | Asian/Asian British |  |
|  | Black, Black British, Caribbean or African |  |
|  | Mixed or multiple ethnic group |  |
|  | Other ethnic group |  |
|  | Prefer not to say |  |
| How would you describe your current employment status? | In full-time paid work |  |
|  | In part-time paid work |  |
|  | Retired |  |
|  | On leave/out of work due to illness or disability |  |
|  | Other, please specify: |  |
|  | Prefer not to say |  |
| What is your current or previous occupation if retired? Please be specific. For example if you are/were in retail, please indicate what you sell/sold. | | |
| What is your marital status? | Married |  |
|  | Single |  |
|  | In a relationship |  |
|  | Divorced |  |
|  | Separated |  |
|  | Widowed |  |
|  | Prefer not to say |  |
| What is your highest educational qualification? | No educational qualifications |  |
|  | O grades, O levels, GCE/GCSEs |  |
|  | Highers, advanced highers, A levels |  |
|  | Vocational qualification (e.g. SVQ, NVQ, SCOTVEC) |  |
|  | Degree (e.g., BA, BSc) |  |
|  | Masters degree (e.g. MSc, MBA) |  |
|  | PhD degree |  |
|  | Professional qualification (e.g. CAEW, CIIA) |  |
|  | Other, please specify: | |
|  | Prefer not to say |  |
| What are your current living arrangements? | Live alone |  |
|  | Live with partner/spouse/children |  |
|  | Live with other adults |  |
|  | Other, please specify: | |
| In general, would you say that your health is... | Excellent |  |
|  | Very good |  |
|  | Good |  |
|  | Fair |  |
|  | Poor |  |
| The next few questions will ask for your views about your health. Answer each question by choosing just one answer. If you are unsure how to answer a question, please give the best answer you can.  The following questions are about activities you might do during a typical day. Does your health now limit you in these activities? If so, how much? | | |
| Moderate activities such as moving a table, pushing a vacuum cleaner, bowling, or playing golf | Yes, limited a lot |  |
|  | Yes, limited a little |  |
|  | No, not limited at all |  |
| Climbing several flights of stairs | Yes, limited a lot |  |
|  | Yes, limited a little |  |
|  | No, not limited at all |  |
| During the past 4 weeks, have you had any of the following problems with your work or other regular daily activities as a result of your physical health? | | |
| Accomplished less than you would like | Yes |  |
|  | No |  |
| Were limited in the kind of work or other activities | Yes |  |
|  | No |  |
| During the past 4 weeks, have you had any of the following problems with your work or other regular daily activities as a result of any emotional problems (such as feeling depressed or anxious)? | | |
| Accomplished less than you would like | Yes |  |
|  | No |  |
| Did work or activities less carefully than usual | Yes |  |
|  | No |  |
| During the past 4 weeks, how much did pain interfere with your normal work (including work outside the home and housework)? | Not at all |  |
|  | A little bit |  |
|  | Moderately |  |
|  | Quite a bit |  |
|  | Extremely |  |
| These questions are about how you have been feeling during the past 4 weeks. For each question, please give the one answer that comes closest to the way you have been feeling. How much of the time during the past 4 weeks… | | |
| Have you felt calm and peaceful? | All of the time |  |
|  | Most of the time |  |
|  | A good bit of the time |  |
|  | Some of the time |  |
|  | A little of the time |  |
|  | None of the time |  |
| Did you have a lot of energy? | All of the time |  |
|  | Most of the time |  |
|  | A good bit of the time |  |
|  | Some of the time |  |
|  | A little of the time |  |
|  | None of the time |  |
| Have you felt down-hearted and blue? | All of the time |  |
|  | Most of the time |  |
|  | A good bit of the time |  |
|  | Some of the time |  |
|  | A little of the time |  |
|  | None of the time |  |
| During the past 4 weeks, how much of the time has your physical health or emotional problems interfered with your social activities (like visiting friends, relatives, etc.)? | All of the time |  |
|  | Most of the time |  |
|  | Some of the time |  |
|  | A little of the time |  |
|  | None of the time |  |
| Do you have any care in place? If no, do you have any plans to put care in place? | | |
| Do you currently use telecare? I.e. pendant alarm? | | |
| Have you used telecare in the past? | | |
| Do you have any health conditions? If so, please list your health conditions. | | |

Now we will go through some statements about your thoughts and feelings. Please tick the box to confirm which description best describes your experience of each over the last 2 weeks. Tick only one box for each statement.

|  | None of the time | Rarely | Some of the time | Often | All of the time |
| --- | --- | --- | --- | --- | --- |
| I've been feeling optimistic about the future |  |  |  |  |  |
| I've been feeling useful |  |  |  |  |  |
| I've been feeling relaxed |  |  |  |  |  |
| I've been feeling interested in other people |  |  |  |  |  |
| I've had energy to spare |  |  |  |  |  |
| I've been dealing with problems well |  |  |  |  |  |
| I've been thinking clearly |  |  |  |  |  |
| I’ve been feeling good about myself |  |  |  |  |  |
| I've been feeling close to other people |  |  |  |  |  |
| I've been feeling confident |  |  |  |  |  |
| I've been able to make up my own mind about things |  |  |  |  |  |
| I've been feeling loved |  |  |  |  |  |
| I've been interested in new things |  |  |  |  |  |
| I've been feeling cheerful |  |  |  |  |  |

Now the next set of questions are about how you have been feeling in the past week. Tick the box beside the reply that is closest to how you have been feeling in the past week. Don’t take too long over your replies: your immediate is best. Tick only one box in each section.

| I feel tense or 'wound up': | | I feel as if I am slowed down: | |
| --- | --- | --- | --- |
| Most of the time |  | Nearly all the time |  |
| A lot of the time |  | Very often |  |
| From time to time, occasionally |  | Sometimes |  |
| Not at all |  | Not at all |  |
| I still enjoy the things I used to enjoy: | | I get a sort of frightened feeling like 'butterflies' in the stomach: | |
| Definitely as much |  | Not at all |  |
| Not quite so much |  | Occasionally |  |
| Only a little |  | Quite often |  |
| Hardly at all |  | Very often |  |
| I get a sort of frightened feeling as if something awful is about to happen: | | I have lost interest in my appearance: | |
| Very definitely and quite badly |  | Definitely |  |
| Yes, but not too badly |  | I don’t take as much care as I should |  |
| A little, but it doesn’t worry me |  | I may not take quite as much care |  |
| Not at all |  | I take just as much care as ever |  |
| I can laugh and see the funny side of things: | | I feel restless as I have to be on the move: | |
| As much as I always could |  | Very much indeed |  |
| Not quite so much now |  | Quite a lot |  |
| Definitely not so much now |  | Not very much |  |
| Not at all |  | Not at all |  |
| Worrying thoughts go through my mind: | | I look forward with enjoyment to things: | |
| A great deal of the time |  | As much as I ever did |  |
| A lot of the time |  | Rather less than I used to |  |
| From time to time, but not too often |  | Definitely less than I used to |  |
| Only occasionally |  | Hardly at all |  |
| I feel cheerful: | | I get sudden feelings of panic: | |
| Not at all |  | Very often indeed |  |
| Not often |  | Quite often |  |
| Sometimes |  | Not very often |  |
| Most of the time |  | Not at all |  |
| I can sit at ease and feel relaxed: | | I can enjoy a good book or radio or TV program: | |
| Definitely |  | Often |  |
| Usually |  | Sometimes |  |
| Not often |  | Not often |  |
| Not at all |  | Very seldom |  |

The next few questions are about feelings of loneliness. Indicate how often each of the statements below is descriptive of you. Tick only one box for each statement.

|  | Never | Rarely | Sometimes | Always |
| --- | --- | --- | --- | --- |
| How often do you feel that you lack companionship? |  |  |  |  |
| How often do you feel left out? |  |  |  |  |
| How often do you feel isolated from others? |  |  |  |  |
| How often do you feel that you are no longer close to anyone? |  |  |  |  |
| How often do you feel that your relationships with others are not meaningful? |  |  |  |  |
| How often do you feel that no one really knows you well? |  |  |  |  |
| How often do you feel that people are around you but not with you? |  |  |  |  |
| How often do you feel that you are isolated from others? |  |  |  |  |
| How often do you feel that you lack companionship? |  |  |  |  |
| How often do you feel that you are in tune with the people around you? |  |  |  |  |
| How often do you feel that you can find companionship when you want it? |  |  |  |  |
| How often do you feel that there are people you can talk to? |  |  |  |  |
| How often do you feel that you have a lot in common with the people around you? |  |  |  |  |
| How often do you feel that you are in touch with those you care about? |  |  |  |  |
| How often do you feel that you have someone to talk to? |  |  |  |  |
| How often do you feel that you have a sense of companionship with others? |  |  |  |  |
| How often do you feel that you have a good friend to talk to? |  |  |  |  |
| How often do you feel that your relationships are satisfying? |  |  |  |  |
| How often do you feel that you are not close to anyone? |  |  |  |  |
| How often do you feel that you are not part of a group of friends? |  |  |  |  |

The final part of the survey is about quality of life. Tick only one box for each statement. Please indicate to what extent you experience the following:

| My age prevents me from doing the things I would like to |  |  |  |  |
| --- | --- | --- | --- | --- |
| I feel that what happens to me is out of my control |  |  |  |  |
| I feel free to plan for the future |  |  |  |  |
| I feel left out of things |  |  |  |  |
| I can do the things I want to do |  |  |  |  |
| Family responsibilities prevent me from doing what I want to do |  |  |  |  |
| I feel that I can please myself what I do |  |  |  |  |
| My health stops me from doing things I want to |  |  |  |  |
| Shortage of money stops me from doing the things I want to do |  |  |  |  |
| I look forward to each day |  |  |  |  |
| I feel that my life has meaning |  |  |  |  |
| I enjoy the things that I do |  |  |  |  |
| I enjoy being in the company of others |  |  |  |  |
| On balance, I look back on my life with a sense of happiness |  |  |  |  |
| I feel full of energy these days |  |  |  |  |
| I choose to do things that I have never done before |  |  |  |  |
| I am satisfied with the way my life has turned out |  |  |  |  |
| I feel that life is full of opportunities |  |  |  |  |
| I feel that the future looks good for me |  |  |  |  |
