# Supplementary material for: Using a Proactive Telecare System to Support Independence, Health, and Well-Being in Older Adults: Feasibility Randomized Controlled Trial
Source: JMIR Form Res. 2025 Dec 31;9:e82152. doi: 10.2196/82152 (PMC12755204; doi:10.2196/82152)
Supplement: Multimedia Appendix 2 [file formative-v9-e82152-s002.docx]

Supplementary File 2. Qualitative Interview Guide

Introduction

In this interview I am interested in two key things:

- What it was like for you to take part in the research
- What it was like for you to use proactive telecare

Taking part in the research

- What interested you to take part in the study? What has it been like taking part in this research?
- How did you feel about being allocated into two groups? (randomised)
- Do you feel that all the information you were given at the beginning of the study was clear? Was it an appropriate time to discuss the study? Was there anything about the study that was unclear/you had concerns about?
- How did you find the courtesy call? Were they useful, or not? Would you have liked the researcher to check in more?
- How have you found completing the questionnaires with the researcher? How was the timing for this/method of collection?

Experiences of OKEachDay

- Which device did you have? Telephone or touchscreen?
- Thinking about your experience of using proactive telecare, what did you hope the device would do to help you live independently? Did using proactive telecare meet your expectations?
  - Did you feel safer using the technology?
  - Did it fit with your daily life? Perception of self?
- How did you feel about using it?
  - What did you like about proactive telecare
  - What did you not like about it?
- Was it easy or hard to set up/use? How did you find integrating it into your routine?
  - Did you experience any challenges in using it?
- What was it like engaging with the technology on a daily basis? What impact, if any did it have on supporting independent living?
- What was your experience of the telephone support? Did you forget to press? How was the support if you did forget? What impact, if any did it have on supporting independent living?
- Were your primary contacts contacted at any point? If so, how was this experience?
- Did you use the reminder functionality of proactive telecare? If so, how was this experience?
- During the trial, did you do anything differently in your life? In terms of living independently.
- Did you see any impacts from using proactive telecare? What was it about the device that helped/hindered these impacts?
- What do you think about the topics we asked about in the questionnaire (i.e. loneliness, anxiety, depression, quality of life)? Is there another impact that you think is important to people that we should be collecting information about in this research?

Ending

Was there anything else you would like to tell me?

Thank you
